# VEXAS Syndrome for the laboratory physician: a case report

**DOI:** 10.3389/fimmu.2026.1809632

**Published:** 2026-05-06

**Authors:** Chaoying Chen, Miaomiao Chen, Tieqiao Chen, Yong Chen, Lingzhi Liu, Yixi Zhu, Xiaoming Yi, Shuguang He

**Affiliations:** 1 Department of Laboratory Medicine, The First Affiliated Hospital of Hunan Traditional Chinese Medical College (Hunan Provincial Directly Affiliated Hospital of Traditional Chinese Medicine), Zhuzhou, China; 2 Department of Radiology, Zhuzhou Central Hospital, Zhuzhou, China; 3 Department of Radiology, The First Affiliated Hospital of Hunan Traditional Chinese Medical College (Hunan Provincial Directly Affiliated Hospital of Traditional Chinese Medicine), Zhuzhou, China

**Keywords:** autoinflammatory disease, bone marrow cells, case report, early diagnosis, laboratory personnel, vacuoles, VEXAS syndrome

## Abstract

**Introduction:**

VEXAS (Vacuoles, E1 enzyme, X-linked, Autoinflammatory, Somatic) syndrome represents a recently identified systemic condition, and its diagnosis and management carry significant clinical ramifications. Despite advancements in understanding the clinical features and pathological mechanisms associated with this syndrome, challenges related to underdiagnosis persist. To date, there have been no reported large-scale prospective studies on this syndrome, and effective, sustained treatment strategies remain undeveloped, contributing to the poor overall prognosis. Consequently, it is crucial to enhance the awareness of this syndrome, delineate its clinical characteristics and associated laboratory indicators, and bolster the capacity for early identification and diagnosis.

**Methods:**

This case report seeks to examine the diagnostic approach to VEXAS syndrome from a laboratory technician’s standpoint, underscoring the necessity for early recognition.

**Results:**

The patient in this case exhibited characteristic clinical manifestations, including fever, rash, arthritis, and hematological irregularities. The diagnosis of VEXAS syndrome was established in conjunction with pertinent laboratory findings.

**Discussion:**

Through this report, we aim to advance the understanding of VEXAS syndrome and enhance the proficiency of laboratory physicians in recognizing the condition in their routine practice, thereby facilitating early diagnosis and treatment for patients and improving overall prognostic outcomes.

## Introduction

VEXAS syndrome was first reported in late 2020 and derives its name from the critical characteristics of the disease, which include vacuoles, E1 enzyme, X-linked inheritance, autoinflammatory features, and somatic mutations. The condition is attributed to somatic mutations in the UBA1 gene, an X-linked gene that encodes the ubiquitin-like modifier activating enzyme 1. Specifically, the missense mutations predominantly occurred at codon 41 within exon 3 of the UBA1 gene, resulting in the following variants: c.121A>G leading to p.Met41Val, c.121T>C leading to p.Met41Thr, and c.121A>C leading to p.Met41Leu (1). VEXAS syndrome is associated with a refractory systemic inflammatory state and various hematological disorders (2). Concurrently, patients diagnosed with VEXAS syndrome exhibit a markedly elevated risk of developing malignant neoplasms in comparison to individuals with other autoinflammatory diseases (3). Current treatment strategies primarily involve high doses of corticosteroids, immunomodulatory and anti-inflammatory agents, as well as allogeneic hematopoietic stem cell transplantation (AHSCT). However, there is a notable absence of large-scale prospective trials addressing the therapeutic management of VEXAS syndrome. The diagnosis and treatment of this condition necessitate the collaboration of a multidisciplinary team within clinical practice (4). This case study outlines the process of identifying VEXAS syndrome and summarizes the experiences encountered during the laboratory inspection course. The presentation of this case not only enhances the current body of literature but also serves as a significant resource for frontline laboratory physicians, facilitating a deeper comprehension of this syndrome and aiding in its identification and diagnosis.

## Case presentation

April 11^th^, 2024, a 64-year-old male patient was admitted to the hospital presenting with fever and localized pain in the left leg, which had persisted for seven days. Following admission, the patient persisted in exhibiting a fever of unknown origin, reaching a peak temperature of 39.5 °C. Physical examination revealed edema in the left leg accompanied by localized patchy erythema of the skin. The patient had a prior medical history of venous thrombosis and recurrent polychondritis, diagnosed three years earlier, characterized by subcutaneous nodules of extremities, red swollen swelling of auricles and hands and feet dorsal surfaces, fever, arthralgia, and other systemic symptoms. These manifestations had been effectively managed with prednisone acetate 20mg/d and methotrexate 10mg and folate 10mg once a week therapies. Furthermore, the patient had a medical history of type 2 diabetes mellitus for over two years and was undergoing glucose management with dapagliflozin. There was no reported history of other infections or detrimental lifestyle habits.

**Figure 1:**
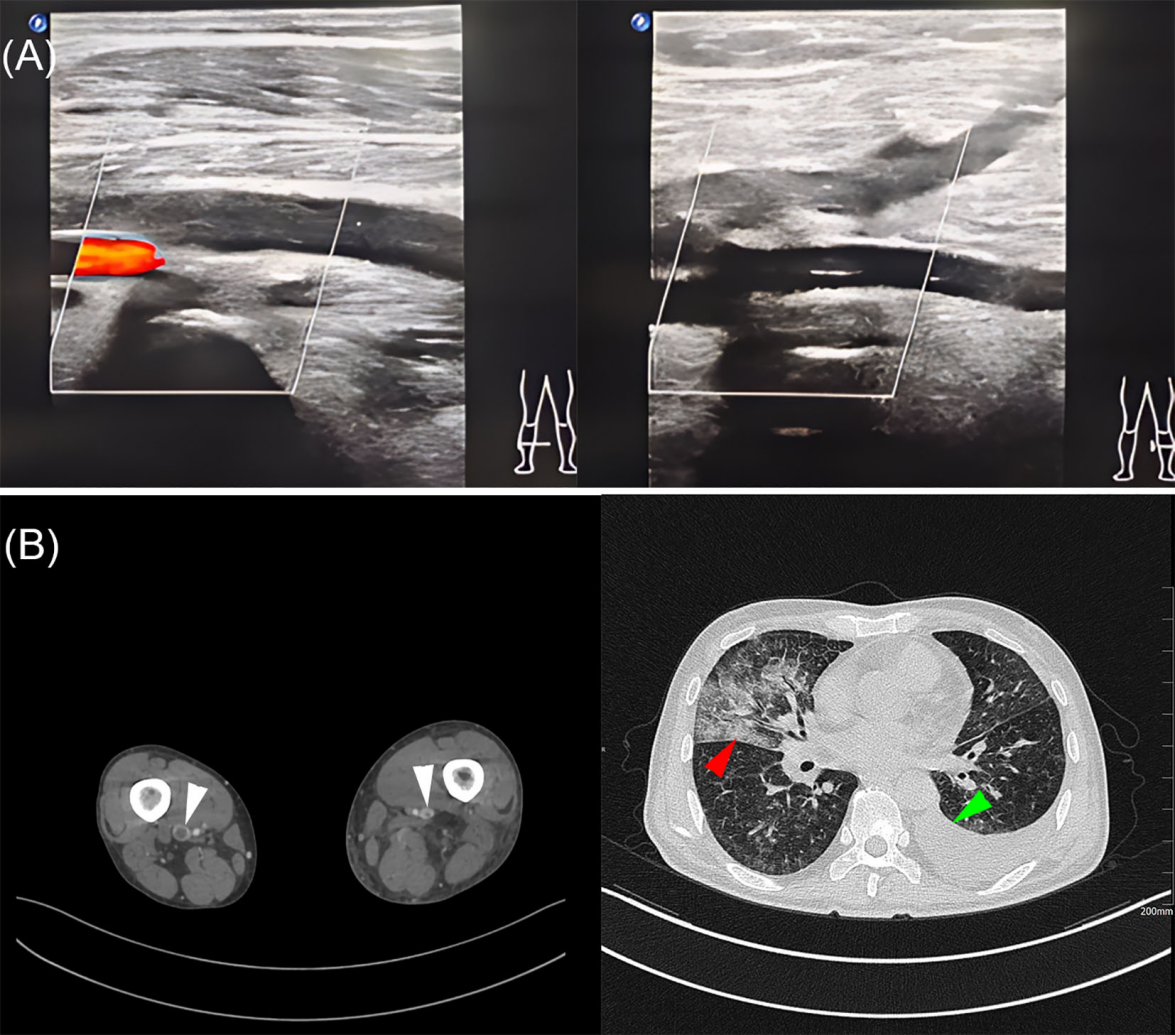
Imageological examination for this patient. **(A)** DVT in both lower extremities by color doppler ultrasound. **(B)** Imaged by CT, white arrowhead indicates thrombus, red arrowhead indicates pulmonary inflammation and green arrowhead indicates hydrothorax.

Upon admission, diagnostic imaging was performed, including color doppler ultrasound and computed tomography (CT) of the bilateral lower extremity veins, which demonstrated the presence of deep venous thrombosis (DVT) in both lower limbs. Additionally, computed tomography angiography (CTA) of the pulmonary artery identified a left-sided pleural effusion and revealed bilateral pulmonary inflammatory lesions (**Figures 1A, B**). Further enhanced CT imaging was recommended for comprehensive evaluation.

Laboratory analyses indicated the patient had macrocytic anemia, with a hemoglobin concentration of 103 g/L and a mean corpuscular volume (MCV) of 111.7 fL, exceeding the normal range for his age (82–100 fL). Additional investigations suggested a coagulation disorder: D-dimer 3385.00ug/L, prothrombin time 16.90sec, international normalized ratio of prothrombin time 1.59, fibrinogen 6.47g/L and an inflammatory syndrome, as evidenced by a hypersensitive C-reactive protein (hs-CRP) level of 196 mg/L and Interleukin 6 (IL-6) was 76.41 pg/mL. April 15- 17, the pertinent pathogenological examinations, including blood culture, sputum culture, nucleic acid assays for human cytomegalovirus and Epstein-Barr virus, nine respiratory pathogens, as well as the (1, 3)-β-D-glucan and galactomannan assays, yielded negative results.

**Figure 2:**
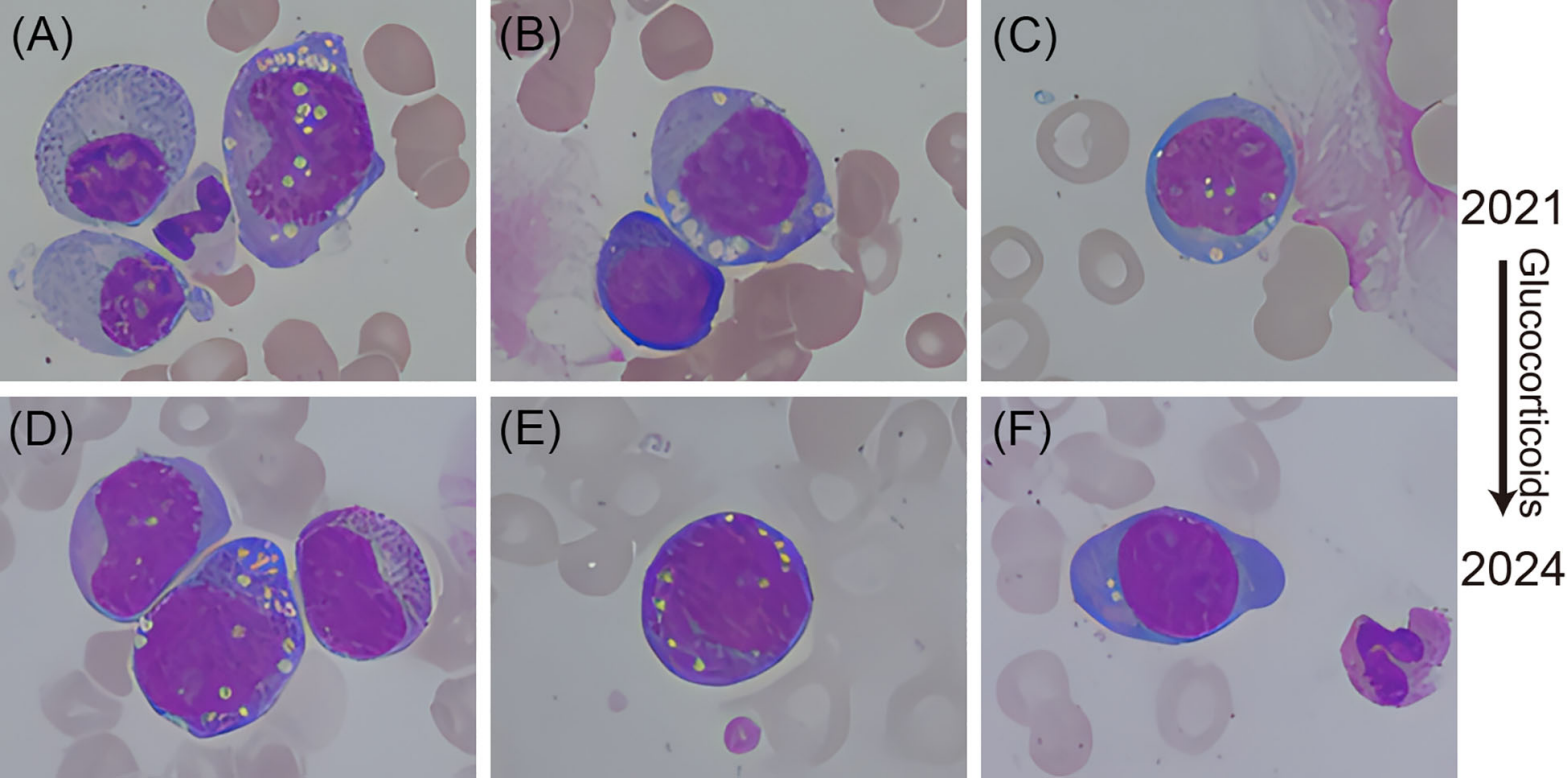
Vacuolation seen in the haematopoietic cells from the bone marrow smear (Wright-Giemsa, 100× objective) of this patient. Top left **(A)**, Top center **(B)**, Bottom left **(D)**, Bottom center **(E)**: Vacuolation in myeloid precursor cells. Top right **(C)**, Bottom right **(F)**: Vacuolation in early erythroid progenitors.

**Figure 3:**
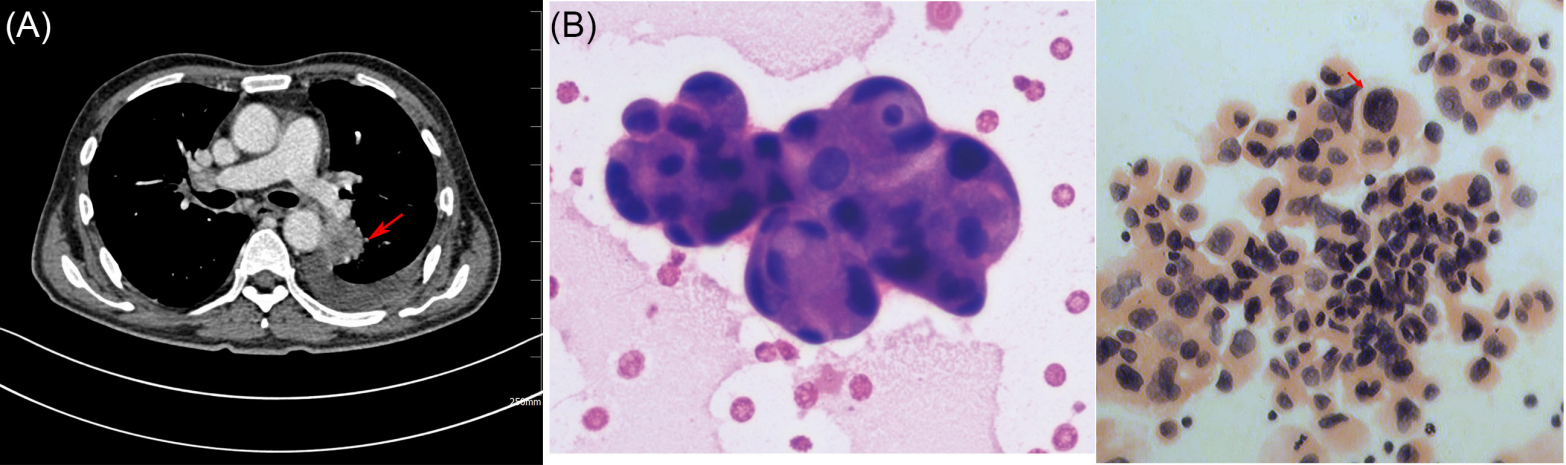
Enhanced CT image of the chest and pathological images obtained from pleural effusion sample. **(A)** The enhanced CT scan reveals a mass located in the lower lobe of the left lung, as denoted by the red arrow. Left **(B)** Malignant tumor cells can be identified in the pleural effusion using hematoxylin and eosin (HE) staining (40× objective). Right **(B)** The DNA ploidy status of malignant tumor cells present in the pleural effusion was assessed using propidium iodide staining (40× objective), as indicated by the red arrow.

A bone marrow biopsy was executed on April 16 and demonstrated active myeloid hyperplasia, with an increased myeloid to erythroid ratio (M:E=8:1) and a notable presence of early myeloid and early erythroid progenitors exhibiting vacuolation (**Figures 2D–F**). The patient experienced a seemingly positive response to anticoagulant therapy. Cefotaxime was administered to address an infection, but its effectiveness was limited, and the patient continued to experience relapsing fever during their hospital stay. On April 17 the contrast-enhanced CT scan demonstrated a tumor located in the posterior segment of the left lower pulmonary lobe, indicative of a high likelihood of lung carcinoma. Concurrently, pathological examination of the pleural effusion detected malignant tumor cells, which immunohistochemical analysis subsequently classified as lung adenocarcinoma cells (**Figures 3A, B**).

We compiled the clinical details of this case and communicated with the clinical team, noting that while myelodysplastic syndrome (MDS) could not be excluded, the patient’s presentation was not typical for MDS and might indicate a rare disease. We recommended genetic testing for genes associated with MDS to confirm the diagnosis. The genetic analysis conducted was a MDS panel encompassing 39 genes associated with MDS, including the UBA1 gene (**Table 1**). Following this discussion, the rheumatology and immunology department reviewed the complex case and noted that the middle-aged male patient had a history of recurrent polychondritis, which responded well to glucocorticoid therapy. During this hospitalization, the patient presented with DVT in both lower extremities, pleural effusion, lung infiltrates, macrocytic anemia, and vacuolated changes in the bone marrow. The possibility of VEXAS syndrome was considered, and genetic testing for UBA1 gene was deemed necessary, which the laboratory medicine team supported. Utilized the patient’s whole blood anticoagulated with EDTA for next-generation sequencing, the results revealed a missense mutation in the UBA1 gene at exon 3 codon 41, resulting in the substitution of methionine with valine (c.121A>G; p.Met41Val), confirming the diagnosis of VEXAS syndrome (**Table 2**). So, the prednisone dosage was increased to 25 mg daily and meropenem was added as antimicrobial treatment since concurrent infection could not be ruled out. Following this treatment adjustment, the patient’s inflammatory markers improved, and their temperature gradually normalized. Actually, the results of this patient’s bone marrow smear indicated the presence of vacuoles in both myeloid and erythroid precursor cells in August 25, 2021 (**Figures 2A–C**). At that time, the patient was diagnosed with relapsing polychondritis and commenced treatment with glucocorticoids.

**Table 1:** The genetic analysis of 39 genes associated with myelodysplastic syndrome.

Catalogue of genetic tests·						
ASXL1	BCOR	BCORL1	CALR	CBL	CEBPA	DDX41
DNMT3A	ETNK1	ETV6	EZH2	FLT3	GATA2	GNB1
IDH1	IDH2	JAK2	KMT2A	KRAS	MPL	NF1
NPM1	NRAS	PHF6	PPM1D	PRPF8	PTPN11	RUNX1
SETBP1	SF3B1	SRSF2	STAG2	STAT3	TET2	TP53
U2AF1	UBA1	WT1	ZRSR2			

**Table 2:** Genetic testing report for tumors of the hematopoietic and lymphoid tissues.

Summary of test results·						
Gene/ transcript number	Nucleotide changes	Amino acid changes	Location of the exons	Types of variation	Proportion of variation/ copy number	Classification of variation
UBA1 NM_003334.4	c.121A>G	p.M4IV	exon3	Missense variants	82.67%	I

At the time of the current admission, the patient was diagnosed with VEXAS syndrome, and vacuoles remained observable in the precursor cells of the bone marrow smear and the quantity of vacuoles was reduced compared to the previous examination (**Figures 2D–F**). Ultimately, the patient’s symptoms were managed, and they were discharged from the hospital on April 25. Subsequently, the patient was admitted to the hospital for treatment of lung cancer on four separate occasions and was pronounced clinically deceased during his hospitalization in the oncology department on February 23, 2025.

## Discussion

VEXAS syndrome is a recently identified condition within the domains of systemic inflammation and hematological disorders, predominantly affecting males over the age of 50. It is associated with a somatic mutation in the UBA1 gene (4). In this particular case, the patient, a middle-aged male, exhibited characteristic symptoms that ultimately led to a definitive diagnosis through genetic testing. Notably, vacuoles were observed in the bone marrow, along with a significant presence of lipid droplet vacuoles in both myeloid and erythroid precursors within bone marrow aspirates. Further examination of the patient’s medical history revealed additional findings, including an inflammatory response, megaloblastic anemia, phlebothrombosis, pulmonary infiltrates, and other related symptoms. Consequently, we recommended that the clinical team conduct genetic analysis for this patient, which ultimately identified a missense mutation in the UBA1 gene, confirming the diagnosis of VEXAS syndrome.

In the context of clinical laboratory physicians identifying VEXAS syndrome, several critical indicators must be considered. Firstly, a significant presence of vacuolated or multivacuolated progenitor cells is observed in bone marrow, particularly with a marked predominance of vacuoles in early progenitors. Secondly, vacuoles are noted in both myeloid and erythroid progenitors, with a greater prevalence in myeloid progenitors. Thirdly, patients typically exhibit macrocytic anemia alongside elevated inflammatory markers. And in this case, a decrease in vacuoles within bone marrow progenitor cells is observed following glucocorticoid therapy (**Figures 2A–C** compared with **Figures 2D–F**), the patient initiated glucocorticoid treatment subsequent to a diagnosis of recurrent polychondritis in August 2021. It is essential to identify these characteristic cells during clinical examinations and to communicate findings promptly with the clinical team to facilitate an accurate diagnosis of the syndrome. However, it is important to note that the absence or low frequency of vacuolation should not preclude the diagnosis of VEXAS syndrome, as atypical UBA1 mutations may be present (5). Research indicated that patients exhibiting vacuolated changes in bone marrow progenitors, along with clinical features such as inflammatory manifestations and responsiveness to glucocorticoid therapy, have a definitive diagnosis rate of 87.5% (6). A study involving 116 patients diagnosed with VEXAS syndrome revealed that unprovoked venous thrombosis represented 35.3% of cases, highlighting it as a significant clinical feature of the syndrome (7). It has been reported that DVT is more common than pulmonary embolism in patients with VEXAS syndrome (8). Furthermore, relevant study indicated that 49% of a cohort of 119 patients with VEXAS syndrome experienced thrombosis, with the majority being unprovoked venous thrombosis. These patients exhibited a tendency for recurrence of thrombosis, even while undergoing anticoagulant therapy (9). In the case presented, the patient experienced a recurrence of DVT, which may be associated with VEXAS syndrome, meanwhile the potential association with lung adenocarcinoma cannot be definitively excluded. In relation to pleural effusion, a study examining the pulmonary manifestations in 45 patients diagnosed with VEXAS syndrome reported that 53% of these individuals exhibited pleural effusion (10), while the presence of pleural effusion in this patient was attributable to lung adenocarcinoma. VEXAS syndrome is linked to hematological malignancies (11), while its concurrence with lung cancer remains relatively uncommon. Current research has identified somatic mutations in the UBA1 gene as a novel oncogenic driver in lung cancer (12). A recent study has documented that, among a cohort of 90 patients diagnosed with VEXAS syndrome, one individual was also diagnosed with lung cancer (3). Consequently, it is plausible to infer a potential association between the lung cancer observed in this patient and the underlying VEXAS syndrome. This underscores the significance of clinical features in the identification of this syndrome. Therefore, it is imperative to identify the characteristic hematopoietic system cells, integrate these findings with the patient’s clinical manifestations, and maintain timely communication with the clinical team to support the diagnosis of VEXAS syndrome. The related laboratory abnormalities and clinical manifestations were outlined in **Table 3**.

**Table 3:** The laboratory abnormalities and clinical manifestations association with VEXAS syndrome.

Laboratory abnormalities				
Hematology	Inflammatory markers	Biochemistry	Coagulation	Bone marrow morphology
LYM#↓	CRP↑	Alb↓	D-Dimer↑	Hyperplastic bone marrow
RBC↓	IL-6↑	A/G↓	PT↑	Vacuoles in erythroid and myeloid precursor cells with a particularly pronounced presence in myeloid progenitor cells
HGB↓	PCT↑	C3↑	PT-INR↑	
MCV↑	ESR↑	C4↑	FIB↑	
MCH↑				
Macrocytic anemia				
Lymphopenia				
Clinical Manifestations				
Unexplained fever (1, 2, 7, 14, 17)		Skin lesions (1, 2, 7, 14, 17)		Eye/ear symptoms (2, 7, 14, 17)
Venous thrombosis (7–9, 14, 17)		Pleural effusion (10, 17)		Lung involvement (1, 2, 7, 10, 14, 16, 17)
Recurrent chondritis (1, 7, 14, 17)		Vasculitis (1, 2, 14, 17)		Weight loss (2, 7, 17)
Cardiac involvement (17)		Inflammatory arthritis (14, 17)		Lung cancer (3)

↓, represents a decrease; ↑, represents an increase; LYM#, Absolute lymphocyte count; RBC, red blood cell; HGB, hemoglobin; HCT, hematocrit; MCV, mean corpuscular volume; MCH, mean corpuscular hemoglobin; CRP, C-reactive protein; IL-6, interleukin-6; PCT, procalcitonin; ESR, erythrocyte sedimentation rate; Alb, albumin; A/G, Albumin-Globulin ratio; PT, prothrombin time; PT-INR, prothrombin time-international normalized ratio; FIB, fibrinogen.

Currently, high-dose glucocorticoids remain the standard first-line treatment for VEXAS syndrome. Based on the pathophysiology of this condition, two primary therapeutic approaches have been proposed: addressing the UBA1-mutant clone and targeting immune and inflammatory pathways. The 2025 consensus guidelines from the American College of Rheumatology (ACR) recommend that, in the management of VEXAS syndrome, agents targeting inflammatory pathways—such as Janus kinase (JAK) inhibitors and interleukin-6 (IL-6) inhibitors—demonstrate superior efficacy compared to conventional disease-modifying antirheumatic drugs (DMARDs), including methotrexate and azathioprine, as well as B-cell-directed therapies like rituximab. Nonetheless, certain study has indicated that cytokine-directed therapies may not effectively reduce glucocorticoid dependency in clinical settings (13). Therapeutic agents aimed at diminishing or eradicating the clonal disease burden, for instance azacitidine, have shown potential benefits for a subset of patients with VEXAS syndrome concomitant with MDS. Furthermore, following thorough hematologic assessment, selected patients with VEXAS may be candidates for AHSCT, which is regarded as a potentially curative intervention (14). However, there is no consistently effective treatment available for all patients with VEXAS syndrome, which significantly contributes to the poor overall prognosis and elevated mortality rates among affected individuals. Relevant report has reported that the five-year mortality rate among patients with VEXAS syndrome ranges from 18% to 40% (15). Consequently, there is a pressing need for large-scale prospective trials to explore effective treatment modalities for this disorder. Related research indicates that the prevalence of VEXAS syndrome is approximately 1 in 4,000 among men over the age of 50 with 100% penetrance (16), categorizing it as a rare condition due to its underdiagnosis. This underdiagnosis is another critical factor contributing to the challenges in early recognition and diagnosis of VEXAS syndrome. The absence of extensive prospective studies underscores the importance of early identification and diagnosis of patients. This highlights the significance of our case report, which aims to enhance awareness among laboratory technicians regarding the syndrome and its characteristic cellular features during laboratory processes. By facilitating timely communication with clinical teams, we can propose effective and targeted interventions, thereby aiding in the early diagnosis and treatment of patients. This, in turn, will enable large-scale clinical research efforts and the pursuit of effective treatment strategies to improve patient outcomes.

This case report is subject to certain limitations, As the relevant imaging and biopsy of the patient’s skin nodules could not be obtained, the characteristic images of skin could not be presented. Furthermore, recent literature indicates that the patient fulfills the majority of the current diagnostic characteristics of VEXAS syndrome (17). Therefore, the imaging and laboratory findings associated with this case retain relevance for the early diagnosis of VEXAS syndrome.

## Conclusion

We present a case study of a patient diagnosed with VEXAS syndrome concomitant with lung adenocarcinoma, reported from the perspective of laboratory physician. This syndrome is not uncommon among middle-aged and elderly male populations. Consequently, we propose a critical alert pattern: in males over 50 years of age presenting with unexplained macrocytic anemia, cytopenias, markedly elevated inflammatory markers, and an absence of definitive diagnosis, consideration should be given to the possibility of VEXAS syndrome. In such cases, genetic testing for mutations in the UBA1 gene is recommended.

## Data Availability

The original contributions presented in the study are included in the article/supplementary material. Further inquiries can be directed to the corresponding authors.

## References

[1] Beck DB Ferrada MA Sikora KA Ombrello AK Collins JC Pei W . Somatic mutations in UBA1 and severe adult-onset autoinflammatory disease. N Engl J Med. (2020) 383:2628–38. doi: 10.1056/nejmoa2026834. PMID: 33108101 PMC7847551

[2] Tosato F Pelloso M Zuin J Basso D . Peripheral blood cells vacuoles in VEXAS syndrome. Am J Hematol. (2023) 98:1663–4. doi: 10.1002/ajh.26931. PMID: 37073682

[3] Gavioli F Caggiano V Sbalchiero J Frassi M Crisafulli F Cavazzana I . VEXAS syndrome and cancer: Insights about a possible “tip of the iceberg”. Ambidirectional data from the international AIDA network registries. Semin Arthritis Rheumatism. (2026) 77:152932. doi: 10.1016/j.semarthrit.2026.152932. PMID: 41619570

[4] Khitri MY Hadjadj J Mekinian A Jachiet V . VEXAS syndrome: An update. Joint Bone Spine. (2024) 91:105700. doi: 10.1016/j.jbspin.2024.105700. PMID: 38307404

[5] Lacombe V Hadjadj J Georgin-Lavialle S Lavigne C Geneviève F Kosmider O . Vacuoles in bone marrow progenitors: VEXAS syndrome and beyond. Lancet Haematology. (2024) 11:e160–7. doi: 10.1016/s2352-3026(23)00375-7. PMID: 38302223

[6] Hines AS Koster MJ Bock AR Go RS Warrington KJ Olteanu H . Targeted testing of bone marrow specimens with cytoplasmic vacuolization to identify previously undiagnosed cases of VEXAS syndrome. Rheumatol (Oxford England). (2023) 62:3947–51. doi: 10.1093/rheumatology/kead245. PMID: 37228016

[7] Georgin-Lavialle S Terrier B Guedon AF Heiblig M Comont T Lazaro E . Further characterization of clinical and laboratory features in VEXAS syndrome: Large-scale analysis of a multicentre case series of 116 French patients. Br J Dermatol. (2022) 186:564–74. doi: 10.1111/bjd.20805. PMID: 34632574

[8] Oo TM Koay JTJ Lee SF Lee SMS Lim XR Fan BE . Thrombosis in VEXAS syndrome. J Thromb Thrombolysis. (2022) 53:965–70. doi: 10.1007/s11239-021-02608-y. PMID: 34817788 PMC8612112

[9] Kusne Y Ghorbanzadeh A Dulau-Florea A Shalhoub R Alcedo PE Nghiem K . Venous and arterial thrombosis in patients with VEXAS syndrome. Blood. (2024) 143:2190–200. doi: 10.1182/blood.2023022329. PMID: 38306657 PMC11143532

[10] Borie R Debray MP Guedon AF Mekinian A Terriou L Lacombe V . Pleuropulmonary manifestations of vacuoles, E1 enzyme, X-linked, autoinflammatory, somatic (VEXAS) syndrome. Chest. (2023) 163:575–85. doi: 10.1016/j.chest.2022.10.011. PMID: 36272567

[11] Sakuma M Blombery P Meggendorfer M Haferlach C Lindauer M Martens UM . Novel causative variants of VEXAS in UBA1 detected through whole genome transcriptome sequencing in a large cohort of hematological Malignancies. Leukemia. (2023) 37:1080–91. doi: 10.1038/s41375-023-01857-5. PMID: 36823397 PMC10169658

[12] Zhang T Joubert P Ansari-Pour N Zhao W Hoang PH Lokanga R . Genomic and evolutionary classification of lung cancer in never smokers. Nat Genet. (2021) 53:1348–59. doi: 10.1038/s41588-021-00920-0. PMID: 34493867 PMC8432745

[13] Turturice BA Fike A Patel BA Groarke EM Gutierrez-Rodriguez F Stonick K . Disease trajectories and glucocorticoid exposure in VEXAS syndrome treated with cytokine-directed therapies. Ann Rheumatic Dis. (2026) 85:370–8. doi: 10.1016/j.ard.2025.05.021. PMID: 40581580 PMC12276827

[14] Mekinian A Georgin-Lavialle S Ferrada MA Savic S Koster MJ Kosmider O . American College of Rheumatology guidance statement for diagnosis and management of VEXAS developed by the International VEXAS Working Group Expert Panel. Arthritis Rheumatol (Hoboken NJ). (2026) 78:509–522. doi: 10.1002/art.43287. PMID: 40787890 PMC12991921

[15] Kötter I Krusche M . VEXAS syndrome: An adult-onset autoinflammatory disorder with underlying somatic mutation. Curr Opin Rheumatol. (2025) 37:21–31. doi: 10.1097/BOR.0000000000001068, PMID: 39470174

[16] Beck DB Bodian DL Shah V Mirshahi UL Kim J Ding Y . Estimated prevalence and clinical manifestations of UBA1 variants associated with VEXAS syndrome in a clinical population. Jama. (2023) 329:318–24. doi: 10.1001/jama.2022.24836. PMID: 36692560 PMC10408261

[17] Groarke EM Turturice B Patel BA Quinn KA Fike A Grayson PC . VEXAS syndrome: A comprehensive review of pathogenesis, clinical spectrum, and therapeutic strategies. Lancet (London England). (2026) 407:637–48. doi: 10.1016/s0140-6736(25)02164-6. PMID: 41520673

